# Property and Function of a Novel Chitinase Containing Dual Catalytic Domains Capable of Converting Chitin Into *N*-Acetyl-D-Glucosamine

**DOI:** 10.3389/fmicb.2022.790301

**Published:** 2022-02-24

**Authors:** Chengyong Wang, Xueman Chen, Ning Zhou, Yan Chen, Alei Zhang, Kequan Chen, Pingkai Ouyang

**Affiliations:** ^1^State Key Laboratory of Materials-Oriented Chemical Engineering, College of Biotechnology and Pharmaceutical Engineering, Nanjing Tech University, Nanjing, China; ^2^Jiangsu Key Laboratory of Marine Bioresources and Environment, Jiangsu Ocean University, Lianyungang, China

**Keywords:** chitin, *Cm*Chi3, dual functional domains, *N*-acetyl-D-glucosamine, hydrolysis pattern, chitinase

## Abstract

A novel multifunctional chitinase (*Cm*Chi3)-encoding gene was cloned from *Chitinolyticbacter meiyuanensis* and actively expressed in *Escherichia coli*. Sequence analysis showed that *Cm*Chi3 contains two glycoside hydrolase family 18 (GH18) catalytic domains and exhibited low identity with well-characterized chitinases. The optimum pH and temperature of purified recombinant *Cm*Chi3 were 6.0 and 50°C, respectively. *Cm*Chi3 exhibited strict substrate specificity of 4.1 U/mg toward colloidal chitin (CC) and hydrolyzed it to yield *N*-acetyl-D-glucosamine (GlcNAc) as the sole end product. An analysis of the hydrolysis products toward *N*-acetyl chitooligosaccharides (*N*-acetyl COSs) and CC substrates revealed that *Cm*Chi3 exhibits endochitinase, *N*-acetyl-β-d-glucosaminidase (NAGase), and transglycosylase (TGase) activities. Further studies revealed that the N-terminal catalytic domain of *Cm*Chi3 exhibited endo-acting and NAGase activities, while the C-terminal catalytic domain showed exo-acting and TGase activities. The hydrolytic properties and favorable environmental adaptations indicate that *Cm*Chi3 holds potential for commercial GlcNAc production from chitin.

## Introduction

Chitin, a linear β-1,4-linked biopolymer of *N*-acetyl-D-glucosamine (GlcNAc), is the second most abundant renewable biomass in nature after cellulose (Kaur and Dhillon, 2015). Chitin is present as a structural constituent in the exoskeletons of arthropods, crustacean shells, and fungal cell walls (Wan and Tai, 2013). About 6–8 million tons of waste crab, shrimp, and lobster shells are produced and discarded annually worldwide, which results in wastage of resources and environmental problems (Yan and Chen, 2015). GlcNAc, which is the monomeric unit of chitin, exhibits many bioactivities and used widely in several fields, such as the food, pharmaceutical, agriculture, and fine chemical industries (Chen et al., 2010). Furthermore, GlcNAc contains nitrogen and is an ideal feedstock for manufacturing diverse intermediates of high value, such as 3-acetamido-5-acetylfuran (3A5AF) (Zang et al., 2021) and 5-hydroxymethylfurfural (5-HMF) (Zhou et al., 2020). Therefore, it would be economically and environmentally beneficial to produce GlcNAc from the abundant chitin resources, such as waste shrimp and crab shells.

Methods involving the conversion of chitin to GlcNAc have been widely explored to date (Husson et al., 2017; Zhang A. et al., 2021). In the commercial production of GlcNAc from chitin, acid hydrolysis at high temperatures is often used. However, with the increased awareness on environmental protection, chemical methods are not preferred as they are associated with pollution (Wei et al., 2017). Therefore, researchers have recently paid more attention to the enzymatic production of GlcNAc by the hydrolysis of chitin using chitin-degrading enzymes. This enzymatic process involves mild production conditions, provides high yields, and results in a product with high bioactivity (Chen et al., 2010).

Chitin-degrading enzymes, which are essential for chitin degradation, can be divided into endochitinase [randomly cleaves chitin at internal sites to release *N*-acetyl chitooligosaccharides (*N*-acetyl COSs)], exochitinase (hydrolyzes chitin oligosaccharides to liberate GlcNAc dimer), and *N*-acetyl-β-glucosaminidase (converts *N*-acetyl COSs to GlcNAc) (Dahiya et al., 2006). To efficiently convert chitin to GlcNAc, a multienzyme system containing at least one chitinase and one *N*-acetyl-β-glucosaminidase (NAGase) is often required. However, using multiple enzymes increases the costs, which limits their industrial application (Zhu et al., 2016). Therefore, identifying a multiple-function chitinase that can degrade chitin to GlcNAc and constructing a single-enzyme catalytic system are likely to be advantageous in reducing the cost and simplifying the conversion process. Several reports have shown that some chitinases have multiple catalytic functions and possess NAGase and chitinase activities (Fu et al., 2014; Zhang et al., 2018). However, these multifunctional enzymes always produce and accumulate GlcNAc dimer in the process of hydrolyzing chitin to prepare GlcNAc.

In our previous study, the chitinolytic bacterium *Chitinolyticbacter meiyuanensis* was isolated, and its extracellular chitin-degrading enzymes were found to efficiently hydrolyze chitin and produce GlcNAc as the sole product (Hao et al., 2011). Furthermore, several key chitinases and their coding genes were identified by combining whole-genome and peptide fingerprint analysis. Of these, a chitinase (ORF3769) containing two GH18 catalytic domains was observed to degrade chitin to GlcNAc without accumulating GlcNAc dimer. To the best of our knowledge, this is the first report on a chitinase with dual GH18 catalytic domains from *Chitinolyticbacter* species. However, its catalytic properties and specific hydrolysis pattern remain unclear.

In this study, the gene encoding ORF3769 (named *Cm*Chi3) was cloned from strain SYBC-H1 and heterologously expressed in *Escherichia coli* BL21(DE3). Sequence analysis, enzymatic properties, and hydrolysis pattern of *Cm*Chi3 were investigated. Furthermore, the individual GH18 catalytic domains of *Cm*Chi3 were studied to determine the hydrolysis mechanism. The findings were expected to provide a detailed understanding of *Cm*Chi3 and its usefulness in directly generating GlcNAc from chitin.

## Materials and Methods

### Chemicals

Chitin was purchased from Aladdin Reagent Co., Ltd. (Shanghai, China). The standards of *N*-acetyl COSs (purity: ≥95%) with a degree of polymerization between 2 and 6 were acquired from Qingdao Bozhi Biotechnology Co., Ltd. (Qingdao, China). Peptone and yeast extract were purchased from Oxoid Co., Ltd. (Beijing, China). All molecular reagents were purchased from TaKaRa Co., Ltd. (Dalian, China). Colloidal chitin (CC) was prepared as described by the method of Gao et al. (2015). Other chemicals and solvents used in this study were purchased from local suppliers and were of analytical grade.

### Strains, Culture Conditions, and Plasmids

*Chitinolyticbacter meiyuanensis* SYBC-H1 (ATCC BAA-2140) used in this study was isolated and cultivated according to our previous study (Zhang et al., 2018). The *E. coli* strains, plasmids, and primers used in this study are listed in Supplementary Table 1. The *E. coli* strains were routinely cultivated aerobically at 37°C in Luria–Bertani (LB) medium (10 g/l tryptone, 5 g/l yeast extract, and 5 g/l NaCl). The transformants were selected on LB plates containing 50 mg/l of ampicillin.

### Identification of Wild-Type *Cm*Chi3 From *Chitinolyticbacter meiyuanensis* SYBC-H1

Wild-type *Cm*Chi3 with chitinase activity was purified using chitinase–glycogen complex precipitation followed by autodigestion of the complex according to the method described in our previous study (Zhang et al., 2018). The corresponding protein (*Cm*Chi3) in the native polyacrylamide gel was sliced for peptide fingerprint analysis using the electrospray ionization quadrupole time-of-flight mass spectrometry (ESI-Q-TOF MS/MS) technique (PROTTECH, Inc., Suzhou, China). These masses were then compared with the theoretical values in Mascot website databases^1^ to discern the amino acid sequences of the peptide fragments. The sequences were then aligned with the genome of strain SYBC-H1 to find *Cm*Chi3 and its coding gene.

### Gene Cloning

The genomic DNA of strain SYBC-H1 was used as the template for polymerase chain reaction (PCR) amplification. The plasmid templates with suitable combinations of primers listed in Supplementary Table 1 were used to generate *CmChi3*, N-terminal GH18 catalytic domain (*Cm*Chi3nGH18), and C-terminal catalytic domain (*Cm*Chi3cGH18). The PCR conditions were as follows: 2 min at 95°C, followed by 35 cycles of 95°C for 20 s, 55–62°C for 20 s, and 72°C for 30–90 s, with a final extension at 72°C for 5 min. After purifying by gel electrophoresis, the PCR products and the vectors were subjected to double digestion using restriction enzymes (*Nde*I and *Eco*RI; *Eco*RI and *Hin*dIII, respectively), followed by ligation using T4 DNA ligase (TaKaRa, Dalian, China). The recombinant plasmids were transformed into *E. coli* DH5α and sequenced by GenScript Biotech (Nanjing, China).

### Sequence Analysis of *Cm*Chi3

Nucleotide and amino acid sequences were analyzed using the Snap Gene v.1.1.3 software and the ExPASy Protparam tool.^2^ The conserved domains and glycoside hydrolase (GH) family classification were identified using the SMART website.^3^ DNA and protein sequence alignments were performed using the National Center for Biotechnology Information (NCBI) server with programs BLASTN and BLASTP,^4^ respectively. Phylogenetic trees were constructed using the neighbor-joining algorithm in MEGA v.7.0 software and assessed using 1,000 bootstrap replications. The presence of a signal peptide and the enzyme location were analyzed using the SignalP v.5.0 server^5^ and the Gneg-mPLoc server v.2.0,^6^ respectively. Protein homologous sequence alignment was performed using ClustalX v.2.1 software and ESPript v.3.0.^7^ The three-dimensional (3D) structure of *Cm*Chi3 was predicted using I-TASSER.^8^

### Gene Expression and Protein Purification

The positive clones were directly screened by colony PCR, transformed into *E. coli* BL21(DE3), inoculated in 10 ml LB medium containing 50 mg/l of ampicillin, and cultured at 37°C in a shaker (200 rpm). Once the optical density (OD_600_) of the culture broth reached 0.6–0.8, isopropyl-β-D-thio-galactopyranoside (IPTG) was added to a final concentration of 1 mM for induction, and the culture was further incubated at 18°C for 20 h. The cells were harvested by centrifugation (6,000 rpm for 10 min at 4°C); after which, they were resuspended in His-tag binding buffer (20 mM Tris–HCl, 500 mM NaCl, 20 mM imidazole, pH 8.0) and lysed by ultrasonication (JY92-IIN, Ningbo Xinzhi Biotechnology, Ltd., Ningbo, China). The cell debris was removed by centrifugation at 8,000 rpm for 30 min at 4°C, and the supernatant was retained as the crude enzyme. The recombinant proteins (chitinases) were purified using a fast protein liquid chromatography (FPLC) system (AKTA Pure 150; GE healthcare Co., Fairfield, CA, United States) with a Ni-nitrilotriacetic acid affinity chromatography (Ni-NTA) column (His Trap FF 5 ml). The target proteins were eluted with elution buffer (20 mM Tris–HCl, 500 mM NaCl, 250 mM imidazole, pH 8.0). The eluted fractions were passed through ultrafiltration tubes of 50 and 10 kDa (Millipore, Burlington, MA, United States) to remove imidazole with 50 mM phosphate buffer saline (PBS, pH 7.0) and concentrate the enzyme solution. All steps in the enzyme purification were performed at 4°C.

### Determination of Enzymatic Activity

Activity assay toward the various substrates was performed using the 3,5-dinitrosalicylic acid (DNS) method (Breuil and Saddler, 1985). Unless otherwise indicated, the reaction mixture containing the suitably diluted enzyme and different polysaccharide substrates at a final concentration of 10 g/l in 100 mM sodium phosphate buffer (pH 6.0) was incubated for 30 min at 50°C. The absorbance was measured at 540 nm, and a standard curve was constructed to determine the amount of reducing sugars produced. One unit of chitinase activity was defined as the amount of enzyme required to produce 1 μmol of reducing sugar at 50°C in 1 min. All chitinase activities were assayed in triplicate, and the average enzyme activity with standard deviation was calculated.

### Determination of Protein Concentration and Molecular Weight

Protein content was determined according to the method of Bradford (1976) using bovine serum albumin as the standard. The specific activity was expressed as units per milligram protein.

The molecular weights and purities of protein samples were analyzed by reductive sodium dodecyl sulfate-polyacrylamide gel electrophoresis (SDS-PAGE) with 20 mM β-mercaptoethanol incubation. A premixed protein marker (TaKaRa Biotechnology Co., Ltd., Nanjing, China) containing 180−, 130−, 95−, 70−, 53−, 40−, 33−, 25−, 17−, and 10-kDa proteins was used as the molecular mass standards.

### Enzymatic Characterization of Recombinant *Cm*Chi3

The optimal temperature for chitinase activity was determined over the range of 25–60°C in 100 mM sodium citrate buffer (pH 6.0) by using 10 g/l CC as the substrate. The thermostability of *Cm*Chi3 was determined by measuring the residual activity after pre-incubation of the purified enzyme in 100 mM sodium citrate buffer (pH 6.0) at 25–60°C in the absence of the substrate for 2 h. The optimal pH for the chitinase activity was assessed in several buffers at 50°C. The following buffers were used: 100 mM sodium citrate buffer, pH 3.0–6.0; 100 mM phosphate buffer, pH 6.0–8.0; Tris–HCl buffer, pH 8.0–9.0; and 0.4 mol/l glycine sodium hydroxide buffer, pH 9.0–10.0. For pH stability, the *Cm*Chi3 was pre-incubated in buffers with different pH at 4°C for 2 h, and the residual activities were determined using 10 g/l of CC as the substrate.

The effects of metal ions on the activity were also determined in this study. Purified *Cm*Chi3 was treated with 10 mM ethylenediaminetetraacetic acid (EDTA) for 5 h at 4°C and then dialyzed with 50 mM PBS (pH 7.0) to remove the EDTA. The activities were assayed as described previously and compared with that of an untreated enzyme solution incubated under similar conditions. For reactivation, the metal-free enzyme was incubated with metal ions (Ca^2+^, Cu^2+^, Co^2+^, K^+^, Na^+^, Al^3+^, Ba^2+^, Ni^2+^, Zn^2+^, Mg^2+^, Mn^2+^, Fe^3+^, and Fe^2+^) at a final concentration of 10 mM for 30 min, and the residual activity was determined. The activity prior to EDTA treatment was used as the control (100%).

### Substrate Specificity and Kinetic Parameters of Recombinant *Cm*Chi3

The substrate specificity was determined using 1% (*w*/*v*) solutions of various polysaccharides [CC, α-chitin powder, β-chitin powder, carboxymethyl cellulose (CMC), hemicellulose powder, and chitosan powder] under the optimum conditions. The amount of reducing sugars released from these polysaccharide substrates was estimated using the DNS method as described previously.

The kinetic parameters against CC under optimum conditions for recombinant *Cm*Chi3 were measured. The initial velocities were determined by incubating 20 μg of purified *Cm*Chi3 with CC of concentrations ranging from 1 to 20 g/l at 50°C in 1-ml reaction system (100 mM sodium citrate buffer, pH 6.0) for 30 min. The amount of reducing sugars released from these substrates was estimated using the DNS method as described previously. *K*_m_ and *V*_max_ values were obtained by Lineweaver–Burk plots (Price, 1985).

### Hydrolysis Pattern of the Recombinant *Cm*Chi3, *Cm*Chi3nGH18, and *Cm*Chi3cGH18 Toward Colloidal Chitin

The reaction system (1 ml of 100 mM sodium citrate buffer, pH 6.0) contained CC (10 g/l) and 20 μg each of purified *Cm*Chi3, *Cm*Chi3nGH18, and *Cm*Chi3cGH18. The reaction was performed at 50°C for various time intervals. The samples were boiled for 5 min to stop the reaction.

### Hydrolysis Pattern of the Recombinant *Cm*Chi3, *Cm*Chi3nGH18, and *Cm*Chi3cGH18 Toward *N*-Acetyl Chitooligosaccharides

Reaction mixtures containing purified enzymes (0.1 μg) and *N*-acetyl COSs (DP 2–6) at a final concentration of 10 g/l were incubated in a 20-μl volume of 100 mM sodium citrate buffer (pH 6.0) at 50°C for various time intervals. The reactions were stopped by boiling for 5 min.

### High-Performance Liquid Chromatography Analysis of the Products

The products were detected using an Agilent 1260 series liquid chromatography system (Agilent Technologies, Santa Clara, CA, United States), according to our previous study (Zhang et al., 2018).

### Nucleotide Sequence Accession Number

The sequence of the chitinase gene *CmChi3* was deposited in the GenBank database under the accession number MZ559373.

## Results and Discussion

### Sequence Analysis of *Cm*Chi3

The *Cm*Chi3 gene (2,943 bp) encodes a protein of 980 amino acids with a calculated molecular mass and the predicted isoelectric (pI) point of 103 kDa and 6.64, respectively. The signal peptide prediction indicated the presence of a putative N-terminal signal peptide (residue 1–33) in the sequence of *Cm*Chi3, which signified that *Cm*Chi3 should be a secretory protein and explained the identification of *Cm*Chi3 in the fermentation broth of strain SYBC-H1.

**FIGURE 1:**
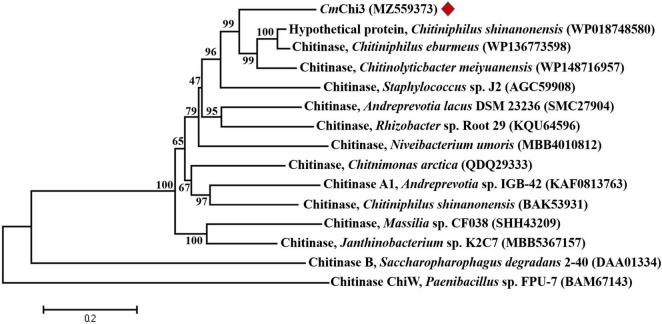
Phylogenetic relationships between chitinase *Cm*Chi3 and other bacterial chitinases from the GH family 18. The phylogenetic tree was constructed using the neighbor-joining algorithm based on the amino acid sequence alignment in MEGA v.7.0. The amino acid sequence of *Cm*Chi3 was aligned with those of the following proteins: GH18 chitinases from *C. shinanonensis* (WP018748580), *C. eburmeus* (WP136773598), *C. meiyuanensis* (WP148716957), *Staphylococcus* sp. J2 (AGC59908), *A. lacus* DSM 23236 (SMC27904), *Rhizobacter* sp. Root 29 (KQU64596), *Niveibacterium umoris* (MBB4010812), *Chitnimonas arctica* (QDQ29333), *Andreprevotia* sp. IGB-42 (KAF0813763), *C. shinanonensis* (BAK53931), *Massilia* sp. CF038 (SHH43209), *Janthinobacterium* sp. K2C7 (MBB5367157), *Saccharopharophagus degradans* 2–40 (DAA01334), and *Paenibacillus* sp. FPU-7 (BAM67143).

According to the Carbohydrate-Active enZYmes (CAZy) database,^9^ chitinolytic enzymes are mainly divided into GH families 18, 19, and 20 based on amino acid sequence similarity. BLASTP analysis showed that *Cm*Chi3 belonged to GH family 18 (GH18) and shared the highest identity (74.52%) with the GH18 chitinase from *Chitiniphilus eburneus* (WP_136773598), followed by a hypothetical protein (73.57%) from *Chitiniphilus shinanonensis* (WP_018748580). However, these coding genes have not been expressed and studied. Among the characterized GH18 chitinases, *Cm*Chi3 showed the highest identity (54.42%) with the GH18 chitinase from *Chromobacterium* sp. C-61 (AAP88583) (Park et al., 2007), followed by the GH18 chitinase (39.48%) from *Bacillus cereus* (AAM48520) (Huang and Chen, 2005) and GH18 chitinase (27.46%) from *Clostridium paraputrificum* (BAA34922) (Morimoto et al., 1999). For chitinases containing two GH18 catalytic domains, ChiW (27.13%) from *Paenibacillus* sp. (BAM67143) (Itoh et al., 2013), and ChiB (25.86%) from *Saccharophagus degradans* 2–40 (DAA01334) (Howard et al., 2004), the putative GH18 chitinase with the highest similarity to *Cm*Chi3 and the verified GH18 chitinase were selected to construct the phylogenic tree, which also showed that *Cm*Chi3 exhibited a low sequence identity with most of the functionally characterized bacterial GH18 chitinases (Figure 1). Multiple alignments of the individual GH18 domains of *Cm*Chi3 with other GH18 chitinases from different sources indicated sequence DxDxE in *Cm*Chi3nGH18 and *Cm*Chi3cGH18 is the highly conserved amino acid sequence among the GH18 family members. DxDxE is a commonly conserved catalytic motif in GH18 family members, in which glutamic acid (E) residue is considered as proton donors in the catalytic process (Supplementary Figure 1; Dai et al., 2021).

**FIGURE 2:**
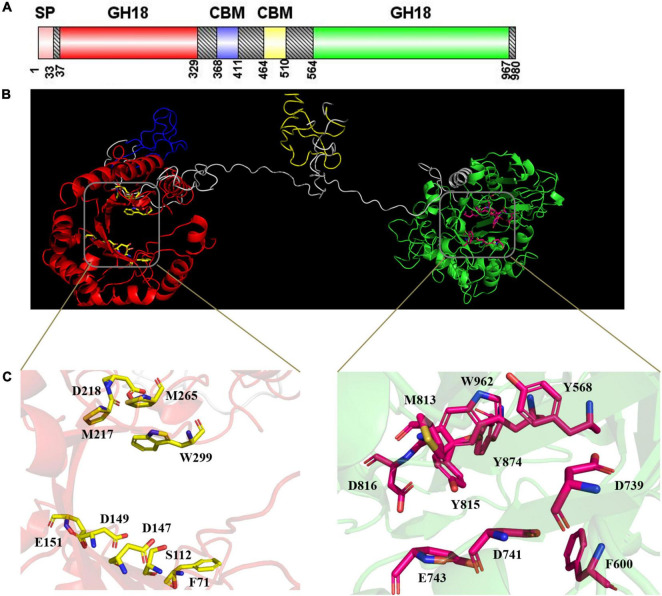
**(A)** Structural features of *Cm*Chi3. A putative signal sequence, two GH18 catalytic domains, and two carbohydrate-binding modules (CBMs) are indicated. This image was generated using IBS Illustrator (Liu et al., 2015). **(B)** The prediction of the 3D structure of *Cm*Chi3. Red indicates Glyco_18 domain; blue indicates CBM; yellow indicates CBM; green indicates Glyco_18 domain; gray indicates unknown region. **(C)** The active sites of *Cm*Chi3. D147, D149, and E151 were the active residues of the N-terminal catalytic domain; D739, D741, and E743 were the active residues of the C-terminal catalytic domain.

Domain structure prediction indicated that *Cm*Chi3 possesses two GH18 catalytic domains (residues 37–329 and 564–967) (Figure 2A). This result is contrary to most GH18 chitinases, which usually possess one GH18 catalytic domain. To date, few microbial chitinases with dual GH18 catalytic domains have been characterized. Meanwhile, accessory binding modules and the catalytic domain can improve the substrate accessibility of chitinases. *Cm*Chi3 also contains two family 5 carbohydrate-binding modules (residues 368–411 and 464–510), which showed that it might possess good binding abilities for the chitinous substrate. As shown in Figure 2, the 3D structure of *Cm*Chi3 was predicted based on the structure model from I-TASSER, which revealed that the amino acid sites F71, S112, D147, D149, E151, M217, D218, M265, W299, Y568, F600, D739, D741, E743, M813, Y815, D816, Y874, and W962 were the active sites of N- and C-terminal catalytic domains, respectively (Supplementary Figure 1). These amino acids possibly form active pockets.

### Cloning, Expression, and Purification of the Recombinant *Cm*Chi3

The gene encoding *CmChi3* without the signal peptide sequence (2,844 bp) was successfully amplified and actively expressed in *E. coli* BL21(DE3). As shown in Supplementary Figure 2, the localization of *Cm*Chi3 was in the supernatant (lane 1) of the cell-free extract of the recombinant *E. coli* BL21(DE3)-(pCold I-*CmChi3*), indicating that *Cm*Chi3 was actively expressed. The recombinant *Cm*Chi3 with N-terminal His-tag was purified by NTA affinity chromatography (lane 2). The SDS-PAGE analysis showed that purified recombinant *Cm*Chi3 possesses a high purity with an approximate molecular weight of 110 kDa, which agrees with the 99.7 kDa calculated from the amino acid sequence (without signal peptide) containing the His-tag. The recombinant *Cm*Chi3 eluted with 250 mM imidazole from a Ni-NTA resin with a recovery yield of 49.1% (Supplementary Table 3).

### Effects of Temperature and pH on the Enzymatic Activity and Stability of Recombinant *Cm*Chi3

**FIGURE 3:**
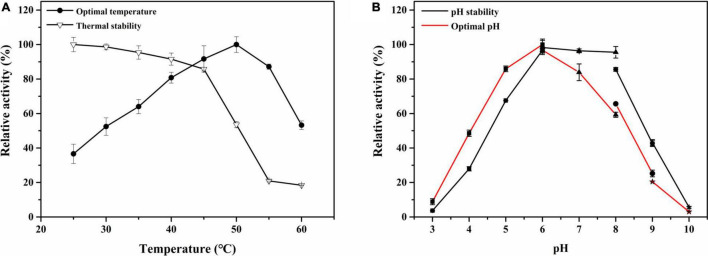
The effect of pH and temperature on the activity and stability of *Cm*Chi3. **(A)** Optimal temperature and thermal stability of the recombinant *Cm*Chi3. The optimum temperature was determined at different temperatures (25–60°C) in 100-mM sodium citrate (pH 6.0) (solid rounds). The residual activity was measured in 100-mM sodium citrate (pH 6.0) after the enzyme was treated for 2 h at different temperatures (open triangles) to determine the thermostability. **(B)** Optimal pH and pH stability of the recombinant *Cm*Chi3. The optimal pH was determined in various buffers within the pH range of 3.0–10.0 [filled square (■), sodium citrate buffer (pH 3.0–6.0); filled triangle (▲), phosphate buffer (pH 6.0–8.0); filled circle (●), Tris–HCl buffer (pH 8.0–9.0); and filled star (★), 0.4 mol/l glycine sodium hydroxide buffer (pH 9.0–10.0)] (red line). The enzyme was incubated at 4°C for 2 h with various pH buffers (black line) to determine pH stability, and the residual activities were measured.

The effect of temperature on *Cm*Chi3 activity was studied. The results showed that recombinant *Cm*Chi3 exhibited maximum activity at a temperature of around 50°C (Figure 3A). This result is comparable to that of some bacterial chitinases, such as CsChiE from *C. shinanonensis* (50°C) (Rani et al., 2020) and PbChi70 from *Paenibacillus barengoltzii* (55°C) (Yang et al., 2016). Few chitinases with dual GH18 domains are ChiW from *Paenibacillus* sp. (50°C) (Itoh et al., 2014), Tk-ChiA from *Thermococcus kodakaraensis* KOD1 (85°C) (Tanaka et al., 1999), and chitinase P1724 from a wetland soil metagenome (40°C) (Dai et al., 2021). Additionally, *Cm*Chi3 was stable and retained more than 90% activity for 2 h at temperatures below 40°C, but it was unstable at temperatures above 50°C.

The effects of pH on the recombinant *Cm*Chi3 were determined in the range of pH 3.0–10.0. As shown in Figure 3B, *Cm*Chi3 exhibited an optimum activity around pH 6.0. Meanwhile, the enzymatic activity of *Cm*Chi3 dropped more than 50% at pH ≤ 4.0 and pH ≥ 8.5 (Figure 3B). This result is similar to that of other bacterial chitinases, such as CsChiE from *C. shinanonensis* (pH 6.0) (Rani et al., 2020) and spChiD from *Serratia proteamaculans* (pH 6.0) (Purushotham and Podile, 2012). However, the optimum pH of chitinases from *Microbispora* sp. V2 (pH 3.0) (Nawani et al., 2002), *Chitinibacter* sp. GC72 (pH 6.8) (Gao et al., 2015), and *Paenibacillus pasadenensis* NCIM 5434 (pH 10.0) (Loni et al., 2014) was different from *Cm*Chi3. With regard to pH stability, *Cm*Chi3 retained more than 60% of its activity after storage at pH 5.0–8.5 for 2 h. This result is comparable to those of other chitinases from *C. meiyuanensis*, including *Cm*Chi1 (pH 5.2–8.2) (Zhang et al., 2018) and *Cm*NAGase (pH 4.0–8.5) (Zhang et al., 2020a).

### Effects of Metal Ions on the Activity of Recombinant *Cm*Chi3

The effects of metal ions on *Cm*Chi3 activity were investigated. All counter-ions of the used metal ions were Cl^–^. As shown in Table 1, EDTA did not affect the enzymatic activity, which indicates that *Cm*Chi3 is non-metal dependent. The activity was strongly inhibited by Cu^2+^, Ni^2+^, and Fe^3+^. The presence of Fe^2+^, Mg^2+^, Ba^2+^, and Na^+^ had a strengthening effect on *Cm*Chi3 activity. Previous works also have shown that Cu^2+^, Ni^2+^, and Fe^3+^ inhibit the activity of chitinases. For example, the chitinase ChiW from *Paenibacillus* sp. was inhibited by Cu^2+^ and Fe^3+^ (Itoh et al., 2014). The chitinase ChiA-Ba43 from *Bacillus altitudinis* KA15 was inhibited by Ni^2+^ (Asmani et al., 2020).

**TABLE 1:** Effects of metal ions on the activity of recombinant *Cm*Chi3.

Metal ions	Chemicals	Concentration (mM)	Relative activity (%)
No addition	–	–	100
Ca^2+^	CaCl_2_	10	98.8 ± 1.3
Co^2+^	CoCl_2_	10	88.9 ± 0.2
Cu^2+^	CuCl_2_⋅2H_2_O	10	25.2 ± 4.0
K^+^	KCl	10	106.2 ± 2.5
Na^+^	NaCl	10	118.1 ± 4.0
Al^3+^	AlCl_3_	10	71.7 ± 0.7
Ba^2+^	BaCl_2_	10	158.5 ± 0.3
Zn^2+^	ZnCl_2_	10	82.9 ± 2.9
Mg^2+^	MgCl_2_	10	116.2 ± 0.3
Mn^2+^	MnCl_2_	10	77.8 ± 0.9
Fe^2+^	FeCl_2_	10	135.7 ± 4.6
Fe^3+^	FeCl_3_	10	44.0 ± 5.0
Li^+^	LiCl	10	98.6 ± 3.4
Ni^2+^	NiCl_2_	10	63.0 ± 0.3
EDTA	EDTA	10	104.3 ± 0.9

### Substrate Specificity and Kinetic Parameters of Recombinant *Cm*Chi3

The hydrolysis ability of *Cm*Chi3 toward various insoluble polysaccharide substrates was investigated. Of the substrates tested (Table 2), CC was most effectively hydrolyzed by the *Cm*Chi3 with a specific activity of 4.1 U/mg, similar to the chitinase with dual catalytic domains, such as ChiW with a specific activity of 5.2 U/mg (Itoh et al., 2014). The activity was higher than that of most other reported chitinases, including chitinase from *Streptomyces speibonae* TKU048 (0.32 U/mg) (Thi Ngoc et al., 2019), chitinase CHI from *Paenibacillus chitinolyticus* (0.75 U/mg) (Liu C. et al., 2020), and Tk-ChiA from *T. kodakaraensis* KOD1 (2.4 U/mg) (Tanaka et al., 2001), but lower than that of *Px*Chi52 from *Paenibacillus xylanexedens* (16 U/mg) (Zhang W. et al., 2021) and *Cm*Chi1 from *C. meiyuanensis* (15.3 U/mg) (Zhang et al., 2018). *Cm*Chi3 displayed low activities toward α-chitin (0.02 U/mg) and β-chitin powder (0.3 U/mg). No activity was found toward CMC, hemicellulose, and chitosan (Table 2).

**TABLE 2:** Substrate specificity of *Cm*Chi3.

Substrate	Specific activity (U/mg)
CC	4.1 ± 0.4
α-Chitin powder	0.02 ± 0.01
β-Chitin powder	0.3 ± 0.02
CMC	ND*
Hemicellulose	ND*
Chitosan	ND*

*Activity was not detected.

Furthermore, the kinetic parameters of recombinant *Cm*Chi3 toward CC were investigated (Supplementary Table 2). The [s]-velocity plots of CC are shown in Supplementary Figure 3. The *K*_m_, *k*_cat_, and *k*_cat_/*K*_m_ values were determined to be 7.53 ± 0.78 mg/ml, 9.08 ± 0.36 s^–1^, and 1.2 ± 0.11 ml/s/mg for CC (Supplementary Table 2).

### Hydrolysis Pattern of the Recombinant *Cm*Chi3

**FIGURE 4:**
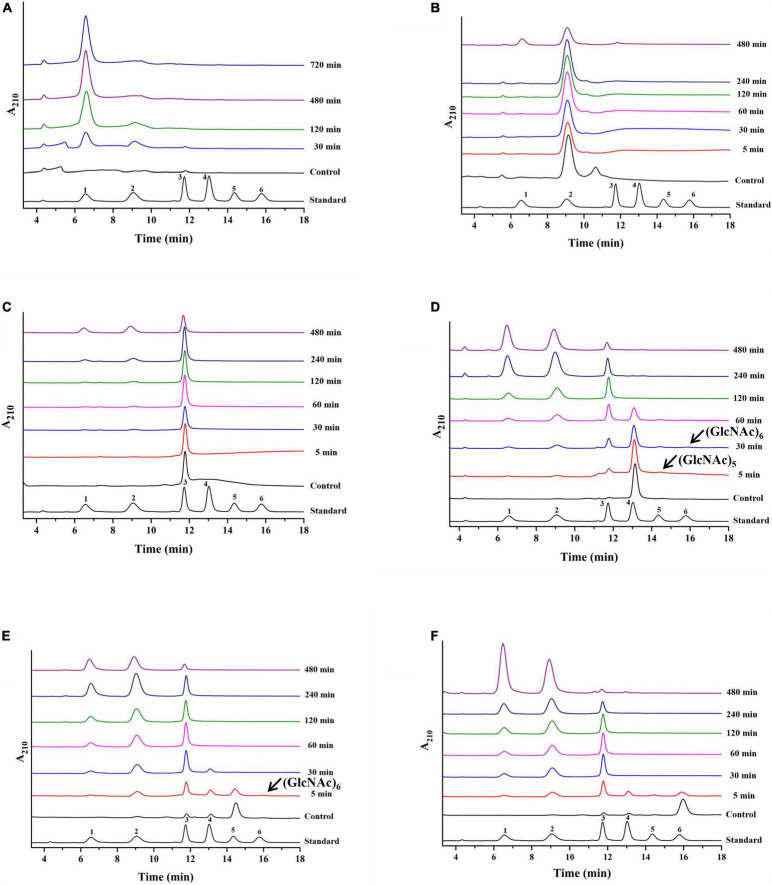
Hydrolysis pattern of *Cm*Chi3 toward CC and *N*-acetyl COSs. The reactions containing 20 μg *Cm*Chi3 and 10 g/l CC or 0.1 μg *Cm*Chi3 and 10 g/l (GlcNAc)_2–6_ were performed in sodium citrate buffer (pH 6.0) at 50°C. Aliquots were withdrawn at different time intervals and analyzed by HPLC. **(A–F)** The hydrolysis products from CC and (GlcNAc)_2–6_. Numbers 1–6 represent GlcNAc to (GlcNAc)_6_.

Colloidal chitin and *N*-acetyl chitooligosaccharides (DP 2–6) were used as hydrolytic substrates to evaluate the hydrolysis pattern of *Cm*Chi3. Our previous study confirmed that *Cm*Chi3 can completely convert CC solely to GlcNAc when using a high enzyme concentration (50 μg) (Zhang et al., 2020b). In this study, a low concentration of enzyme (20 μg) was used to study the specific intermediates of CC hydrolysis. As shown in Figure 4A, *Cm*Chi3 hydrolyzed CC to mainly produce GlcNAc, with a small amount of (GlcNAc)_2_ and (GlcNAc)_3_ in 30 min. Then, it yielded GlcNAc as the sole end product after 120 min, suggesting that *Cm*Chi3 possesses endochitinase and NAGase activities. Previously reported multifunctional chitinases *Cm*Chi1 from *C. meiyuanensis* (Zhang et al., 2018) and *Pb*Chi74 from *P. barengoltzii* (Fu et al., 2014) hydrolyzed CC to produce (GlcNAc)_2_ in the initial hydrolysis stage. Also, (GlcNAc)_2_ needed a longer reaction time to achieve full conversion to GlcNAc, which is different from our observation.

For (GlcNAc)_2–6_, *Cm*Chi3 slowly hydrolyzed (GlcNAc)_2_ into GlcNAc, showing that *Cm*Chi3 possesses NAGase activity. This result is similar to that of other studies (Liu Y. et al., 2020). However, (GlcNAc)_2_, (GlcNAc)_2–3_, (GlcNAc)_2–4_, and (GlcNAc)_2–5_ were each released from (GlcNAc)_3_, (GlcNAc)_4_, (GlcNAc)_5_, and (GlcNAc)_6_, respectively, which suggested that *Cm*Chi3 has endocleavage activities. Additionally, small amounts of (GlcNAc)_5–6_ and (GlcNAc)_6_ were observed when using (GlcNAc)_4_ and (GlcNAc)_5_ as substrates, respectively. This finding suggests that *Cm*Chi3 has a weak TGase activity toward (GlcNAc)_4_ and (GlcNAc)_5_ (Figure 4). Conclusively, *Cm*Chi3 is a multifunctional chitinase possessing endochitinase, NAGase, and TGase activities.

### Hydrolysis Pattern of the Two GH18 Catalytic Domains, *Cm*Chi3nGH18 and *Cm*Chi3cGH18

Recently, several reports have shown that a single enzyme containing more than one catalytic domain may be particularly powerful in degrading polysaccharides, such as a cellulase from the thermophilic bacterium *Caldicellulosiruptor bescii*, which consists of GH9 and GH48 domains (Brunecky et al., 2013). Additionally, an enzyme from the actinobacterium *Jonesia denitrificans* contains AA10 lytic polysaccharide monooxygenase (LPMO) and GH18 catalytic domain (Mekasha et al., 2020). According to previous reports, chitinases with dual catalytic domains often possess different catalytic activities. For example, in the chitinase Tk-ChiA from *T. kodakaraensis* KOD1 (Tanaka et al., 2001) and chitinase ChiB from *S. degradans* 2–40 (Itoh et al., 2013), the N-terminal and C-terminal GH18 domains possessed exo- and endo-activities, respectively. In another study, Larsbrink et al. (2016) showed that chitinase ChiA from *Flavobacterium johnsoniae* comprised two GH18 domains, one of which was proposed to have a predominantly endo-activity. In contrast, another one was predicted to have an exo-acting activity (Larsbrink et al., 2016).

To determine the hydrolysis pattern of individual GH18 domains of *Cm*Chi3, N-terminal (*Cm*Chi3nGH18) and C-terminal (*Cm*Chi3cGH18) GH18 catalytic domains were successfully expressed and purified (Supplementary Figure 4). Then, the hydrolysis pattern of *Cm*Chi3nGH18 and *Cm*Chi3cGH18 toward CC and (GlcNAc)_2–6_ was analyzed. As shown in Supplementary Figure 5, *Cm*Chi3nGH18 hydrolyzed CC to produce (GlcNAc)_1–3_ (Supplementary Figure 5A) and hydrolyzed (GlcNAc)_3–6_ to (GlcNAc)_1–2_, (GlcNAc)_1–3_, (GlcNAc)_1–4_, and (GlcNAc)_1–5_, which showed that *Cm*Chi3nGH18 possesses endochitinase activity. Additionally, *Cm*Chi3nGH18 converted (GlcNAc)_2_ into GlcNAc (Supplementary Figure 5B), thus exhibiting NAGase activity.

*Cm*Chi3cGH18 hydrolyzes CC solely into (GlcNAc)_2_ (Supplementary Figure 5G). For (GlcNAc)_2–6_, (GlcNAc)_2_ could not be hydrolyzed. When using (GlcNAc)_3_ as the substrate, the products were (GlcNAc)_2_ and GlcNAc. (GlcNAc)_4_ was mainly converted into (GlcNAc)_2_. When using (GlcNAc)_5–6_ as the substrate, (GlcNAc)_2_, (GlcNAc)_3_, and (GlcNAc)_4_ were generated (Supplementary Figure 5). These results showed that the enzyme has exochitinase activity. Also, *Cm*Chi3cGH18 synthesized higher *N*-acetyl-COSs (GlcNAc)_5–6_ from (GlcNAc)_4_ and (GlcNAc)_6_ from (GlcNAc)_5_, showing that it possesses TGase activity. This result also explains why little (GlcNAc)_3_ was observed from (GlcNAc)_4_ and (GlcNAc)_6_, (GlcNAc)_4_ was generated from (GlcNAc)_5_, and (GlcNAc)_5_ was obtained from (GlcNAc)_6_.

The above results showed that *Cm*Chi3nGH18 displayed endochitinase and NAGase activities and *Cm*Chi3cGH18 showed exochitinase and TGase activities. The results agree with those of *Cm*Chi3.

## Conclusion

In this study, a novel chitinase (*Cm*Chi3) containing dual GH18 catalytic domains and two carbohydrate-binding modules from *C. meiyuanensis* was cloned, heterologously expressed in *E. coli*, and biochemically characterized. The analysis of the hydrolysis products revealed that *Cm*Chi3 is a multifunctional chitinase, which possesses exochitinase, NAGase, and TGase activities. Further studies on the individual catalytic domains of *Cm*Chi3 showed that N-terminal catalytic domain exhibited endo-acting and NAGase activities, while the C-terminal domain showed exo-acting and TGase activities. The multidomain structure of *Cm*Chi3 is expected to produce GlcNAc from chitin. This work provides a reference to chitinase with multifunctional domains and GlcNAc enzymatic production from chitin.

## Data Availability Statement

The datasets presented in this study can be found in online repositories. The names of the repository/repositories and accession number(s) can be found in the article/Supplementary Material.

## Author Contributions

CW: writing-original draft preparation, formal analysis, and data curation. XC: investigation and methodology. NZ and YC: methodology. AZ: funding acquisition and writing-review and editing. KC and PO: conceptualization and supervision. All authors contributed to the article and approved the submitted version.

## Conflict of Interest

The authors declare that the research was conducted in the absence of any commercial or financial relationships that could be construed as a potential conflict of interest.

## Publisher’s Note

All claims expressed in this article are solely those of the authors and do not necessarily represent those of their affiliated organizations, or those of the publisher, the editors and the reviewers. Any product that may be evaluated in this article, or claim that may be made by its manufacturer, is not guaranteed or endorsed by the publisher.

## Footnotes

1
http://www.matrixscience.com

2
http://web.expasy.org/protparam/

3
http://smart.embl-heidelberg.de/

4
http://blast.ncbi.nlm.nih.gov/Blast.cgi

5
http://www.cbs.dtu.dk/services/SignalP/

6
http://www.csbio.sjtu.edu.cn/bioinf/Gneg-multi/

7
http://espript.ibcp.fr/ESPript/cgi-bin/ESPript.cgi

8
https://zhanglab.ccmb.med.umich.edu/I-TASSER/

9
www.cazy.org/
