# Supplementary material for: Property and Function of a Novel Chitinase Containing Dual Catalytic Domains Capable of Converting Chitin Into N-Acetyl-D-Glucosamine
Source: Front Microbiol. 2022 Feb 24;13:790301. doi: 10.3389/fmicb.2022.790301 (PMC8908422; doi:10.3389/fmicb.2022.790301)
Supplement: Supplementary file 1 [file Data_Sheet_1.docx]

Supporting Information for

**Property and function of a novel chitinase containing a dual catalytic domainscapable of converting chitin into *N*-acetyl-D-glucosamine**

Chengyong Wang^a^,Xueman Chen^a^, NingZhou^a^, Yan Chen^a^,AleiZhang^a,b,^*,Kequan Chen^a^, PingkaiOuyang^a^

^a^*State Key Laboratory of Materials-Oriented Chemical Engineering, College of Biotechnology and Pharmaceutical Engineering, Nanjing Tech University, Nanjing 211816, China.*

*^b^Jiangsu Key Laboratory of Marine Bioresources and Environment, Jiangsu Ocean University, Lianyungang, 222005, China.*

*Corresponding author.

E-mail address: zhangalei@njtech.edu.cn.

**Table S1** Strains, plasmids, and primers used in this study.

| Strain, plasmid,  or primer | Description*^a^* | Source |
| --- | --- | --- |
| Strains  *E.coli*DH5α  *E.coli*BL21(DE3)  Strain SYBC-H1 | *ϕ*80d*lac*ZΔM15 Δ(*lacZY-argF*)U169 *deoR recA*1 *endA1 hsdR17*(r_k_^-^ m_k_^+^) *supE44 thi*-1 *gyrA96 relA1*  *relA1*F^-^ *ompThsdS_B_(r_B_^-^m_B_^-^) gal dcm* (DE3)  Wild type, isolated from soil | Invitrogen  Novagen  Novagen  This study |
| Plasmids  pCold I simple  pET-Duet simple  pCold I-*CmChi3*  pET-Duet-*nCmChi3*  pET-Duet-*cCmChi3* | *E.coli*cloning vector; Amp^r^  *E.coli*expression vector,T7 RNA polymerase gene promoter and terminator; Amp^r^  pCold I simple derivate, containing the *Cm*Chi3 gene from SYBC-H1 strain  6.4-kb pET-Duet derivate carrying the n*Cm*Chi3 gene  6.6-kb pET-Duet derivate carrying the c*Cm*Chi3 gene | TaKaRa  Novagen  This study  This study  This study |
| Primers  *CmChi3*-F  *CmChi3*-R  *CmChi3nGH18*-F  *CmChi3nGH18*-R  *CmChi3cGH18*-F  *CmChi3cGH18*-R | 5’GGGAATTCCATATGACCGAGATCGCCCCGTAC 3’  5’ CCGGAATTCGCGGCCGTTGCCCAGCAC 3’  5’ CCGGAATTCGGCGCTGAAAACGCTGAGC 3’  5’ CCCAAGCTTCGCTGGGGTCACCGGTAC 3’  5’ CCGGAATTCGGCTCCGGTCACCCCGGTC 3’  5’ CCCAAGCTTGCCCAGCACCTTGGCAAG3’ | This study  This study  This study  This study  This study  This study |

**Table S2**Kinetic parameters of recombinant *Cm*Chi3.

| *V*_max_ (μmol/(min·L)) | *K*_m_ (mg/mL) | *k*_cat_ (s^-1^) | *k*_cat_/*K*_m_ (mL/s/mg) |
| --- | --- | --- | --- |
| 116.28 ± 4.9 | 7.53± 0.78 | 9.08± 0.36 | 1.2± 0.11 |

**Table S3**Purification summary of recombinant *Cm*Chi3.

| **Purification method** | **Total activity**  **(U)** | **Total protein (mg)** | **Specific activity (U/mg)^a^** | **Purification (fold)** | **Recovery yield (%)** |
| --- | --- | --- | --- | --- | --- |
| Crude enzyme | 103.4 | 517.2 | 0.2 | 0 | 100 |
| Ni-NTA resin | 50.8 | 12.4 | 4.1 | 41.7 | 49.1 |

^a^Enzyme activity was measured in 100 mM sodium phosphate buffer (pH 6.0) at 50℃for30 min using 1% (w/v) of colloidal chitin as substrate.

**
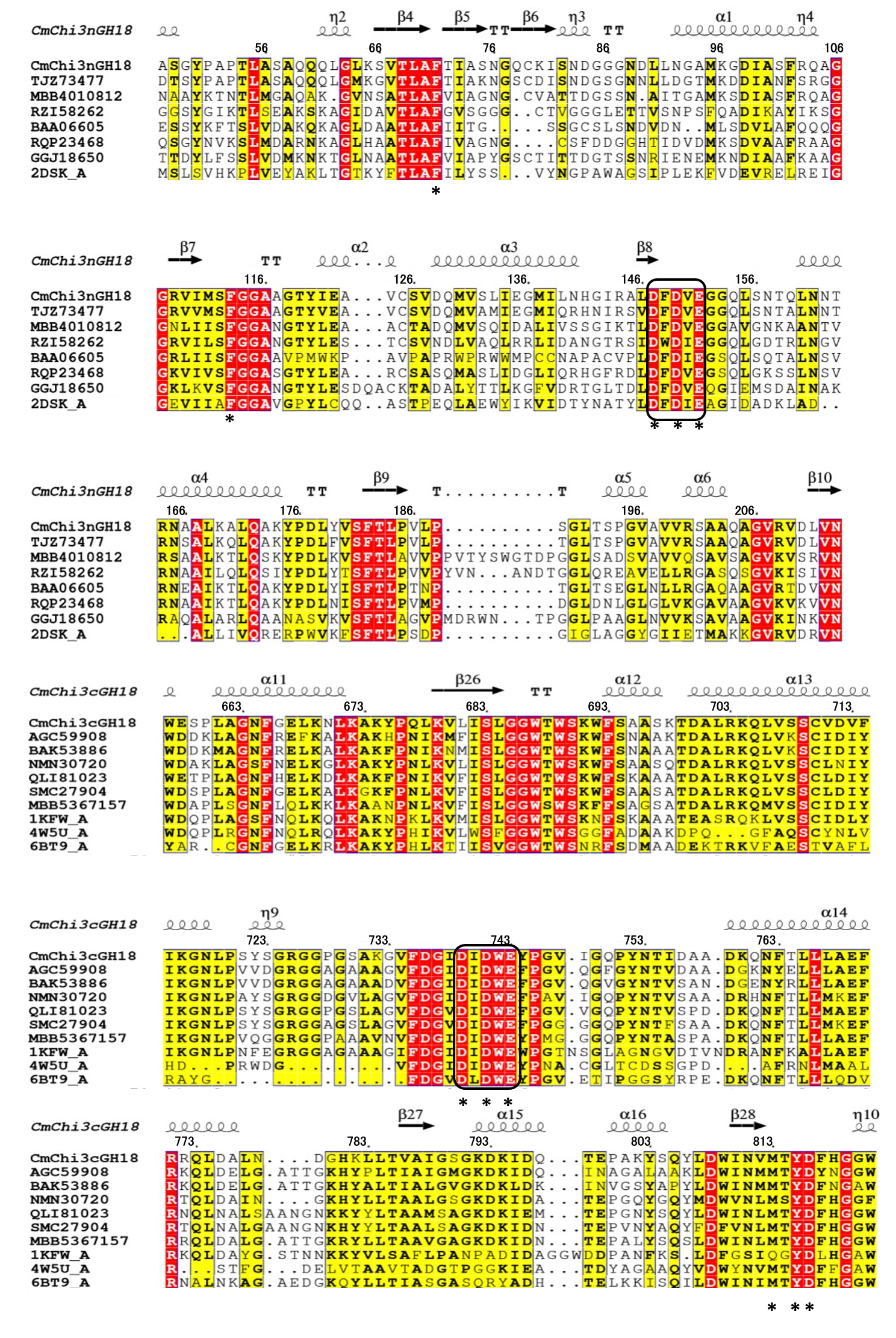
**

Fig. S1. Multiple alignments of the amino acid sequences of GH18 catalytic domain in *Cm*Chi3nGH18 and *Cm*Chi3cGH18 related GH18 family chitinases. The other listed sequences included the chitinases from*Chitiniphiluseburneus* (TJZ73477),*Niveibacteriumumoris* (MBB4010812), *Rubrivivax*sp.(RZI58262), *Aeromonas* sp. 10S-24 (BAA06605),*Geomonas soli* (RQP23468),*Deinococcus roseus*(GGJ18650), *Pyrococcusfuriosus* (2DSK_A); *Staphylococcus* sp.J2 (AGC59908), *Chitiniphilusshinanonensis*(BAK53886), *Rhizobacter*sp. SG490 (NMN30720),*Chitinibacterfontanus* (QLI81023), *Andreprevotialacus* DSM 23236 (SMC27904),*Janthinobacterium*sp. K2C7 (MBB5367157), *Althrobacter* TAD20 (1KFW_A), *Streptomyces thermoviolaceus* (4W5U_A), *Bacillus thuringiensis* (6BT9_A). Yellow represents conserved residues between sequences. The black asterisks represent the conserved catalytic active sites (data not shown completely).

**
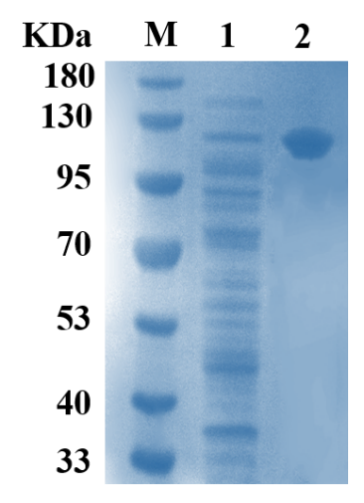
**

Fig. S2. SDS-PAGE analysis of the soluble expression and purification of recombinant *Cm*Chi3. Lane M, protein molecular mass markers; lane 1, the cell lysate of the recombinant expression strain; lane 2, purified *Cm*Chi3 (8 μg) by His-tag affinity chromatography.


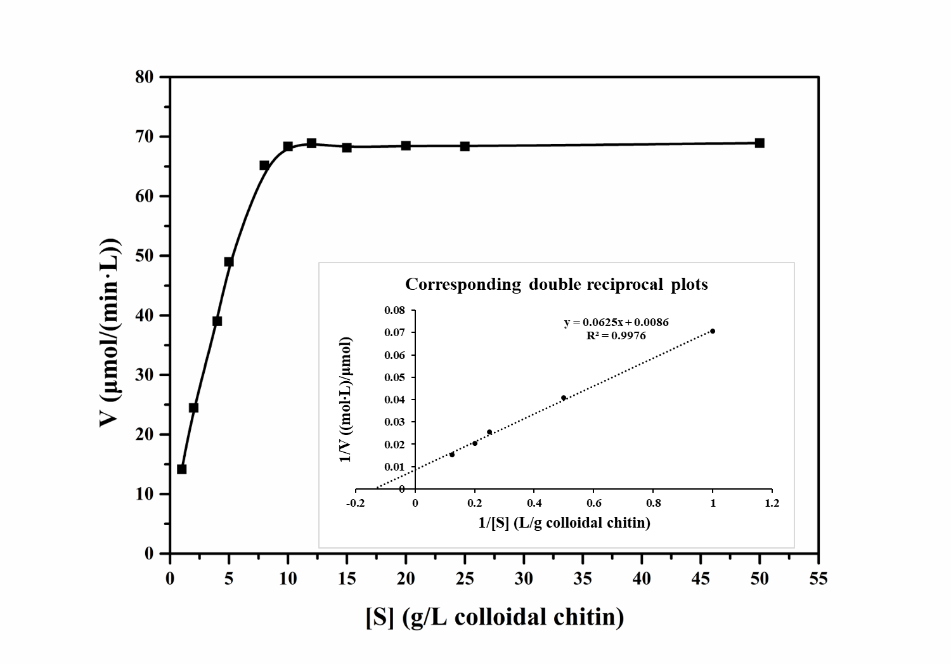

Fig. S3. Determination of *K*_m_ and *V*_max_ of the purified *Cm*Chi3 enzyme from C. *meiyuanensis* strain SYBC-H1 using colloidal chitin as substrate. Chitinase activities were carried out in triplicate and were incubated for 30 min at 50°Cin 100 mM sodium phosphate buffer (pH 6.0) with 1 to 50g/L CC. Inset: corresponding double reciprocal plots of the purified chitinase *Cm*Chi3. The linear regression gives an equation with r^2^ = 0.9976.

**
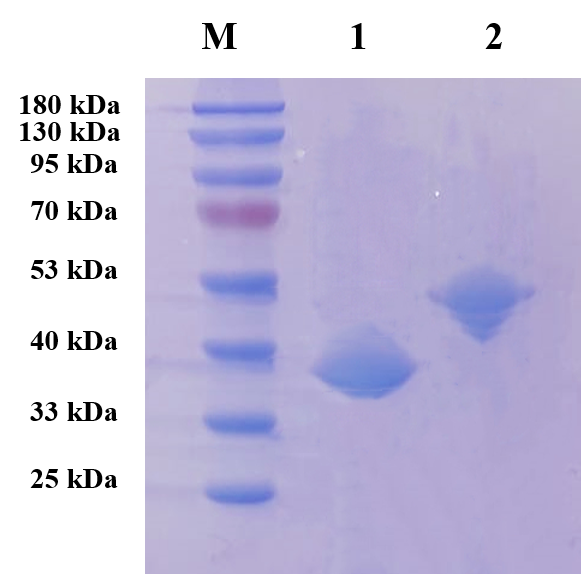
**

Fig. S4. SDS-PAGE analysis of the purificationof recombinant *Cm*Chi3nGH18 and *Cm*Chi3cGH18.Lane M, protein molecular mass markers; lane 1, *Cm*Chi3nGH18 (10 μg)purified by His-tag affinity chromatography.; lane 2, *Cm*Chi3cGH18 (10 μg)purified by His-tag affinity chromatography.

**
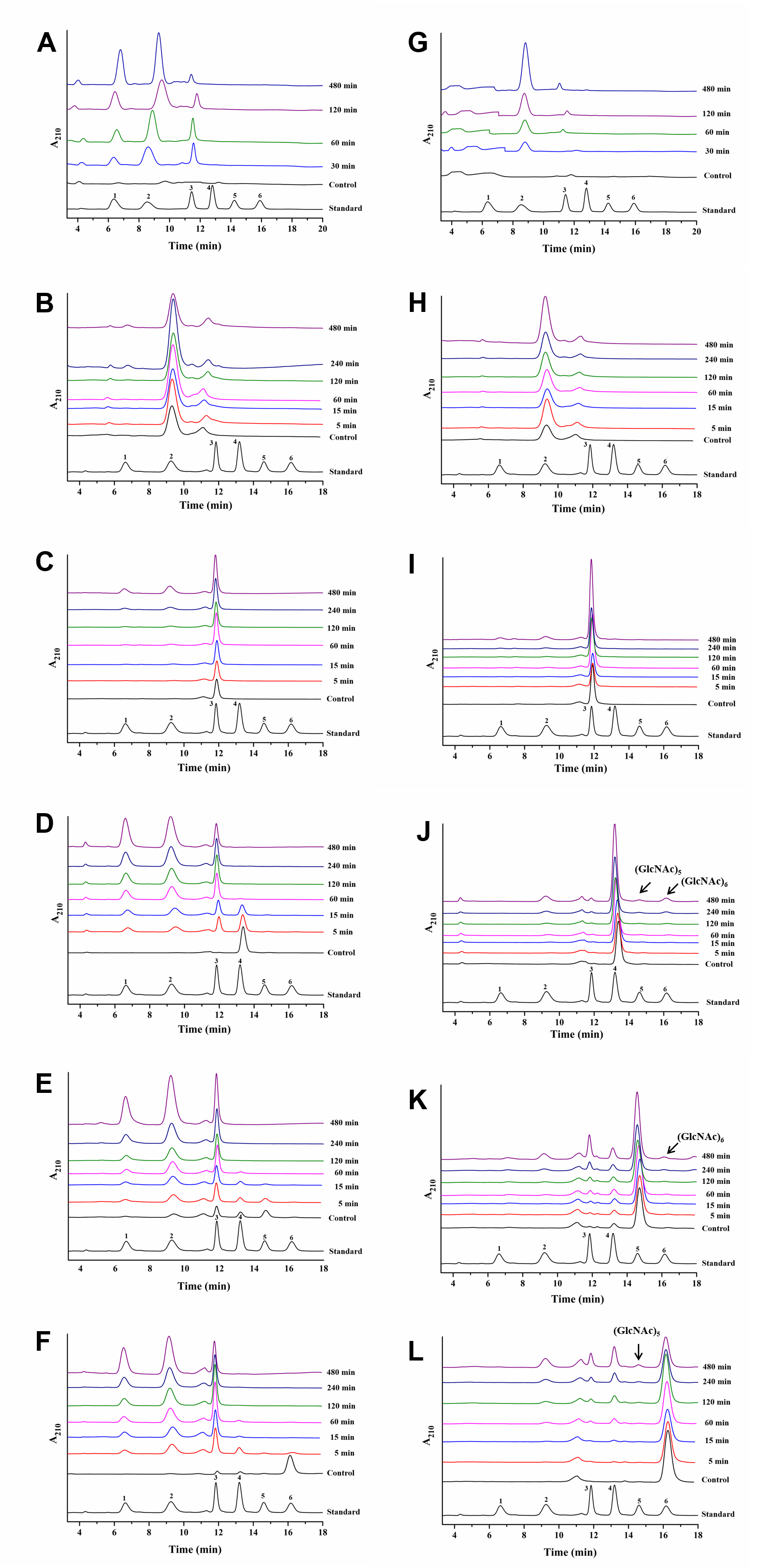
**

Fig. S5. Hydrolysis of *Cm*Chi3nGH18 and *Cm*Chi3cGH18 toward CC and *N*-acetyl COSs. The reactions contained 0.1 μg*Cm*Chi3nGH18, *Cm*Chi3cGH18, and 10 g/L (GlcNAc)_2–6_were performed in sodium citrate buffer (pH 6.0) at 50℃. Aliquots were withdrawn at different time intervals and analyzed by HPLC. *Cm*Chi3nGH18 (A, B, C, D, E, F) and *Cm*Chi3cGH18(G, H, I, J, K, L) showed the hydrolysis products from CC and (GlcNAc)_2–6_, respectively.Numbers 1–6 representGlcNAc to (GlcNAc)_6_.
